# Changes in Predictive Task Switching with Age and with Cognitive Load

**DOI:** 10.3389/fnagi.2017.00375

**Published:** 2017-11-22

**Authors:** Shelly Levy-Tzedek

**Affiliations:** ^1^Recanati School for Community Health Professions, Department of Physical Therapy, Ben-Gurion University of the Negev, Beer-Sheva, Israel; ^2^Zlotowski Center for Neuroscience, Ben-Gurion University of the Negev, Beer-Sheva, Israel

**Keywords:** aging, motor control, predictive control, cognitive load, anticipatory control of movement, negative hysteresis, task switching, switching cost

## Abstract

Predictive control of movement is more efficient than feedback-based control, and is an important skill in everyday life. We tested whether the ability to predictively control movements of the upper arm is affected by age and by cognitive load. A total of 63 participants were tested in two experiments. In both experiments participants were seated, and controlled a cursor on a computer screen by flexing and extending their dominant arm. In Experiment 1, 20 young adults and 20 older adults were asked to continuously change the frequency of their horizontal arm movements, with the goal of inducing an abrupt switch between discrete movements (at low frequencies) and rhythmic movements (at high frequencies). We tested whether that change was performed based on a feed-forward (predictive) or on a feedback (reactive) control. In Experiment 2, 23 young adults performed the same task, while being exposed to a cognitive load half of the time via a serial subtraction task. We found that both aging and cognitive load diminished, on average, the ability of participants to predictively control their movements. Five older adults and one young adult under a cognitive load were not able to perform the switch between rhythmic and discrete movement (or vice versa). In Experiment 1, 40% of the older participants were able to predictively control their movements, compared with 70% in the young group. In Experiment 2, 48% of the participants were able to predictively control their movements with a cognitively loading task, compared with 70% in the no-load condition. The ability to predictively change a motor plan in anticipation of upcoming changes may be an important component in performing everyday functions, such as safe driving and avoiding falls.

## Introduction

With aging, there is often a decline in some aspects of movement control, such as reaction time (Lodha et al., 2016) and explicit motor sequence learning (Bo et al., 2009). There are more postural preparation errors (Verrel et al., 2016), the incidence of falls increases (Fuller, 2000), and so does the variability in motor reactions (Lodha et al., 2016). Potentially, some of these “faults” in motor control stem from a difficulty in predicting upcoming changes in the environment that call for adjusting one’s motor plan (Levy-Tzedek et al., 2011b). That is, it is possible that what is impaired is the ability to *“pre-act”* – to perform a motor action in anticipation of an anticipated change – rather than *re-act* to a change that already took place. Our first goal in the current study was to test the effects of age on the predictive control of movements. Specifically, we tested the effects of age on predictive *switching* between two movement types. It should be noted that the current work does not address *sensory* predictions (Friston, 2010), the effects of age on which were recently reported (Wolpe et al., 2016).

The ability to anticipate changes in the environment and react to them is a key skill in everyday life. Predictive control incorporates a prediction of the future behavior of the system, as opposed to *reactive control*, which responds to the current state of the system. The ability to identify a change in the environment and adapt to it pre-emptively, in a feed-forward manner, rather than rely on the inherently delayed feedback-based response, is an important life skill, as it can prevent injury (as in the case of retracting an arm from a heat source prior to sensing pain) or even avoid life-threatening situations (Porr and Worgotter, 2003; Gilbert and Wilson, 2007). Performing an action in an anticipatory manner improves the speed, the accuracy (Bastian, 2006; Galea et al., 2012) and the efficiency (Fu et al., 2010) of the action. In everyday life, we often prepare for an upcoming change with an appropriate set of motor commands. This phenomenon is particularly apparent with moving one’s gaze in preparation for an upcoming change in movement direction (Grasso et al., 1998), in postural reorganization when preparing to step on surfaces of different texture (MacLellan and Patla, 2006), like sand or snow, or when moving objects of different weights (Sainburg et al., 1999; Pigeon et al., 2013).

Updating a motor command in response to an external stimulus may result in switching between movement types, such as when switching from walking to running. Transitioning between activities is associated with a high risk of falls in the elderly (Rapp et al., 2012; Robinovitch et al., 2013; Woolrych et al., 2014), and it is important to understand what factors contribute to poorly planned transitions.

The great majority of studies on anticipation have studied the ability to *explicitly* predict an upcoming change. However, in everyday life, there are many sensory cues that are perceived implicitly and need to be integrated to form an understanding of changes in the surrounding, such that an appropriate response can be planned. The ability to recognize changes without awareness and respond to them has been termed “a ‘smart’ unconscious” (Cleeremans et al., 1998). An example from everyday life is our **postural control** – i.e., the coordination of muscle activity to stabilize and maintain posture and balance (Gimmon et al., 2015), whether standing, walking, or performing other activities. Although this is a voluntary control process, it is mostly done *in the absence of explicit awareness* of the motor plans generated to maintain balance. And yet, postural sway increases when older adults are asked to perform a working-memory task while maintaining postural balance (Doumas et al., 2009), implying that a cognitive load can interfere with a task that is considered to be implicitly controlled. When healthy individuals know their balance is about to be challenged, e.g., by being pushed or by lifting a load, they perform anticipatory postural adjustments (APAs) (Slijper et al., 2002; Kanekar and Aruin, 2014). Studies on APAs support **the dual-process hypothesis** (also referred to as the parallel-process hypothesis), whereby control of explicit movements (e.g., bending to lift a load) is done separately from the control of implicit movements, which are made to maintain balance during the change in body posture (Slijper et al., 2002).

Another, related, type of anticipatory adjustment has been recorded in object manipulation tasks (Johansson and Flanagan, 2009), as manifest, for example, in grip force variations (Flanagan and Tresilian, 1994; Mawase and Karniel, 2010). For a review on age effects on predictive control of finger force coordination (see Diermayr et al., 2011).

Lastly, predictive control of movement has been demonstrated also in a collaborative task, where two expert improvisers were asked to play the mirror game (Kashi and Levy-Tzedek, 2017), where one person follows the movement of the other, and the small time intervals between the movement arrest times of the two participants indicated the follower was employing a predictive-control strategy (Noy et al., 2011).

Little is known about anticipatory control of implicitly performed movements outside the realms of postural control and grip-force control. We set out to test implicit control of upper arm movements.

We asked whether one element of declined motor ability with age is a decreased ability to “pre-act,” or to perform an implicit change in movement in anticipation of a future change in conditions. To that end, we tested 20 young participants and 20 older participants on a unique task that studies predictive control of movement (Experiment 1). We further asked whether there is a cognitive component to the predictive control of movement, and whether it depends on working-memory resources. To answer that, we ran Experiment 2 on 23 young individuals, who performed the same task, but were subject to a cognitive load half of the time, by performing a parallel computation task. The details of the task for the measurement of predictive control are outlined below.

**FIGURE 1:**
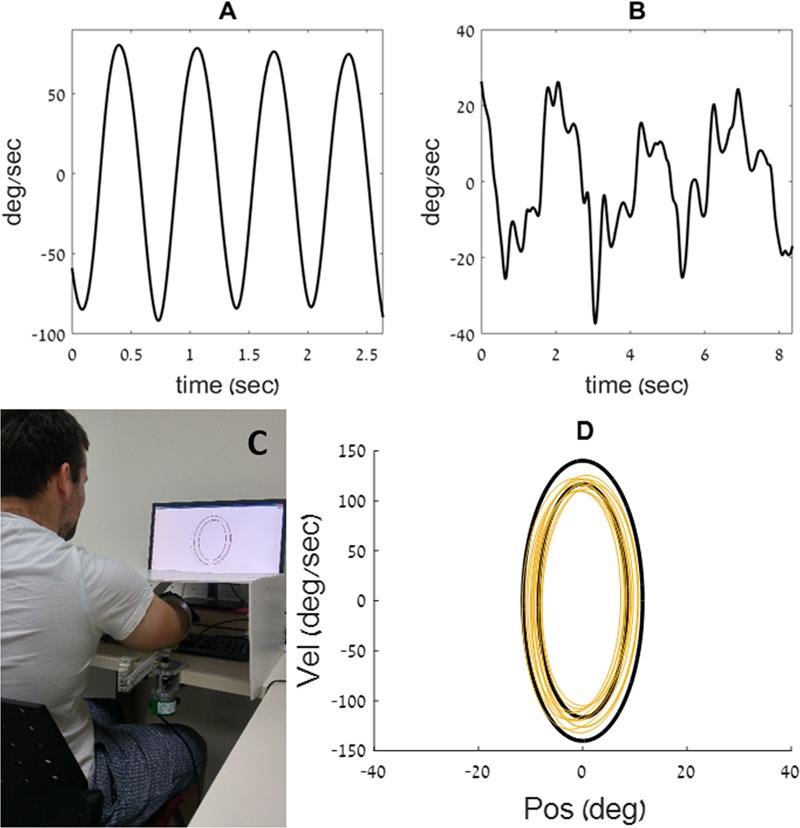
Examples of velocity traces, from the current experiment, recorded during a rhythmic **(A)** and during a discrete **(B)** movement. **(C)** The participant places his arm on the armrest, below an opaque cover. He uses the movements of his forearm to control a cursor on a phase plane, displayed on a computer screen **(D)** example of a training ellipse ‘C’ from the current experiment, shown in black on a phase plane, along with the movement trace of a young participant (in yellow).

In a series of previous studies with healthy participants, we established the classification of repetitive 1-dimensional movements of the forearm (see Materials and Methods) into two distinct movement types – **rhythmic** and **discrete** (Levy-Tzedek et al., 2010) (see **Figures 1A,B**, respectively). These can be conceptualized as the upper-limb equivalents of walking and running, respectively. We found that discrete movements were performed at a low frequency of forearm movement, and rhythmic movements were performed at high forearm frequencies. We then tested how people transition between discrete and rhythmic movements.

We asked participants to perform out-and-back movements with their forearm parallel to the ground at continuously increasing or decreasing frequencies, and studied the transitions between the two movement types (Levy-Tzedek et al., 2011b). We found that the *gradual* change in movement frequency induced an unaware *abrupt* change from a discrete to a rhythmic movement type, or vice versa (for related studies, see also van der Wel et al., 2009; Sternad et al., 2013).

We found a range of intermediate frequencies at which both rhythmic and discrete movements were performed. Surprisingly, within this range of frequencies that enabled both movement types (discrete and rhythmic), we found that switching between the two types of movement was done in an anticipatory fashion: when we asked participants to continuously increase the frequency of their movements, thereby forcing the switch from a discrete to a rhythmic movement type, the switch occurred at a *lower* frequency than the frequency at which they switched from rhythmic to discrete movement types, when the required frequency was decreasing. Rather than maintaining for as long as possible the type of movement with which they started (either rhythmic or discrete), which would be manifested as a “classical hysteresis” pattern, a “reverse hysteresis” pattern emerged from their performance (c.f., Sternad et al., 2013). We interpreted this reverse-hysteresis behavior as indicative of the participants employing anticipatory, or predictive, control of movement (Levy-Tzedek et al., 2011b). Thus, although the participants were not explicitly aware of the fact that they were switching between two movement types, they performed this switch not based on physical (i.e., musculoskeletal) limitations and not in a feedback-based manner, which would have resulted in a classical (positive) hysteresis pattern. Rather, anticipating the upcoming change in task requirements, they were able to “pre-act,” rather than react, by using feed-forward control, with an appropriate change in motor plan. In Experiment 1, we set out to test whether aging affects this ability to “pre-act.” In Experiment 2, we tested whether a cognitive load affects the ability to “pre-act.”

For this study, we specifically chose a task that was previously unfamiliar to our participants, young and old, so that any training effects from past experience would not be a factor in task performance. Furthermore, we employed a task that calls for an *implicit* switch between motor plans – i.e., a switch that is performed without awareness – to eliminate the process of a conscious decision making, and get at the basic pattern of predictive motor control.

We have previously shown that older adults are able to perform a *static* version of the proposed task (Levy-Tzedek et al., 2010, 2011c), where the required frequency remained constant, and did not dynamically change during a trial (see Materials and Methods). Thus, their ability to control the spatiotemporal aspects of their movements has been established.

It is known that some cognitive resources (Park and Reuter-Lorenz, 2009; Cai et al., 2014), including working memory abilities (Gazzaley et al., 2005) decline with age. This decline, in turn, can affect motor behavior (Langan and Seidler, 2011). It has been hypothesized that aging is associated with a shift from automatic movement control to attentional movement control (Seidler et al., 2010), such that movement coordination becomes more purposeful, and requires more cognitive resources. Age-related declines in visuospatial working memory have been associated with reduced motor performance (Bo et al., 2009; Anguera et al., 2011). We hypothesize that the ability to “pre-act” depends on cognitive resources, and therefore expect not to find a significant reverse-hysteresis pattern when an older population performs the task, or when a young population experiences a cognitive load.

If this is indeed the case, it would demonstrate that though the task is implicit (the overwhelming majority of participants are not able to describe they switched between two movement types), performing it ***in a predictive manner*** draws on cognitive resources.

## Materials and Methods

We start by giving an overview of the equipment and methodology that is common to both experiments. We then detail the specifics of each experiment separately.

### Equipment

We used the custom-made experimental apparatus described in Levy-Tzedek et al. (2010, 2011a,b,c), Ben-Tov et al. (2012), and Levy-Tzedek (2017). It is comprised of an arm rest, connected to an encoder that records the flexion/extension angle of the elbow with an accuracy of 0.002° per count at 200 Hz. The arm rest was designed to be as light as possible, to minimize its effect on the natural behavior of the limb. The arm rest was free to rotate around its axis, with no imposed limits on the range of motion. Participants placed their arm on the arm rest, positioned parallel to the table on which it was mounted, and moved their forearm toward and away from their body in a movement similar to that of a windshield wiper. An opaque cover was placed on the table, above the experimental apparatus, such that direct visual information about the arm’s position was precluded. Participants received real-time feedback on their arm’s location via a cursor that appeared on a computer screen that was placed on the table in front of them (see **Figure 1C**).

Participants’ arm was placed in a wrist brace, to prevent movements of the wrist. The forearm flexion/extension movements were one-dimensional, in the horizontal plane. No explicit timing cues were given.

### Protocol

The computer display presented a phase plane, such that position was displayed on the *X*-axis, and velocity on the *Y*-axis (see **Figure 1D**). On that phase plane, a pair of black concentric ellipses created an enclosed doughnut-shaped area, that defined the lower and the upper bounds of the amplitude (on the *X*-axis) and speed (on the *Y*-axis) allowed on the task. The participants controlled a cursor on the phase plane with their arm movements. They were asked to maintain the cursor within the doughnut-shaped area at all times (see yellow trace in **Figure 1D**). Thus, the amplitude, speed and frequency of the movement were prescribed.

The experimental protocol sequence was as follows: the participants started each trial at a neutral position, from which they could comfortably flex and extend their forearm, and this neutral position defined the center of the concentric ellipses (i.e., it is the point of zero position and zero velocity, prior to initiation of the movement).

#### Training

**FIGURE 2:**
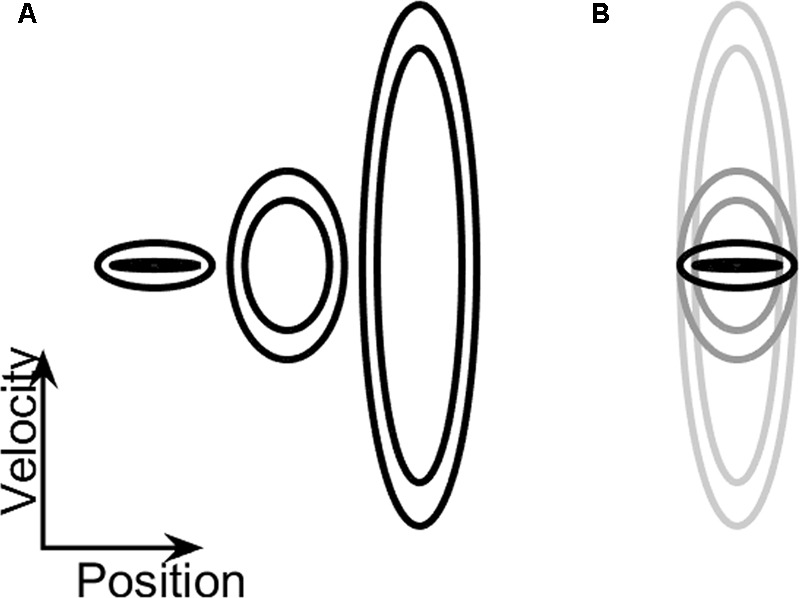
**(A)** Training ellipses. An illustration of the relative size of the three practice ellipses (A, B, and C, from left to right), on which participants trained prior to the testing session. The vertical axis denotes velocity and the horizontal one denotes position. **(B)** Testing ellipses. The enclosed shape on the phase plane was continuously changed during the dynamic task: while the amplitude requirement (i.e., the width of the ellipse along the *x*-axis) remained constant, the speed requirement gradually increased (the ellipse became taller) or decreased (the ellipse became shorter). The ellipse that requires low-frequency movement is marked with a black outline, and the one that requires high-frequency movement is marked with a light-gray outline.

Participants practiced controlling their arm movements such that the cursor on the phase plane stays within the prescribed ellipse. There were three different doughnut sizes on the phase plane during the training session (see **Figure 2A**). Participants were given explicit instructions on how to maintain the cursor between the two ellipses. They were told that the arm movements to the right and to the left controlled the right-left location of the cursor on the screen, and that the height of the cursor depended on the speed of their movement. Once they started practicing, they were given specific instructions, such as “make faster (or wider) movements, in order to stay within the doughnut.” These instructions were given only during the training phase.

On these practice trials, the shape of the doughnut did not change during the trial. These practice ellipses had central frequencies of 0.1 Hz, 0.7 Hz, and 2 Hz (see **Table 1**). The participants were given the option to repeat the practice trials, each lasting 40 s, until they felt comfortable with the task. The order at which these practice ellipses was presented across participants was counterbalanced.

**Table 1:** Target frequency values for each of the three practice ellipses.

Practice ellipse		A	B	C
Frequency (in Hz)	Center	0.1	0.7	2.0
	Range	0.03–0.2	0.5–1	1.6–2.6

Once the participants felt comfortable with this coordinated control of speed and amplitude, the testing phase began.

#### Testing

In the testing phase, task requirements changed dynamically: the height of the enclosed shape on the phase plane either increased, requiring a higher movement speed and frequency, or it decreased, requiring a slower, lower-frequency movement (see **Figure 2B**). Thus, in performing this dynamically changing task, the participants were required to continuously change the frequency of their arm movements. Unbeknownst to them, this was designed to induce an abrupt switch in their motor plan between two distinct movement types: rhythmic (at high frequency, which has a very smooth velocity profile) and discrete [at low frequency, which is highly intermittent (Levy-Tzedek et al., 2011b)]. That is, while the participants were explicitly aware of the continuous change in the shape of the target zone on the screen, and the need to adjust their movement speed accordingly, they were not aware of the switch between the two movement types (discrete and rhythmic) that is necessary in order to successfully complete the task.

Participants performed two blocks of four trials each, with each trial lasting 66.5 s. The two blocks differed in the direction of the gradual change in frequency: either increasing (INC) or decreasing (DEC). This information was conveyed to the participants by the gradually changing vertical extent of the target ellipses on the visual display; the taller were the displayed ellipses, the higher was the required speed and frequency of the forearm movement. The range of ellipse sizes in the testing phase was defined by ellipses A and C in the training phase. That is, the lowest frequency value for movement along the center of the “doughnut” was 0.1 Hz and the highest value was 2 Hz. This translates to a required change in frequency of approximately 0.03 Hz/s over the entire trial. Increasing-frequency trials started at the low frequency, and ended at the high frequency, and decreasing-frequency trials started with the high frequency and ended with the low one (see an illustration of the corresponding changes in ellipse height in **Figure 2B** and Supplementary Video S1). All ellipses defined a target arm amplitude of 20 ± 3°, which corresponded to a visual angle of ∼6°, and remained constant throughout the experiment. The order of presentation of the blocks was counter-balanced across participants.

At the conclusion of the experiment, participants were asked about their experience. They were explicitly asked how many types of movement they performed during the experiment.

Ethical approval for this study was obtained from the Ethics Committee at the Ben-Gurion University of the Negev. All experimental procedures were performed in accordance with this ethical approval.

Below we detail the differences between Experiment 1 and Experiment 2.

### Experiment 1 – Effects of Aging on Predictive Control

#### Participants

A total of 40 individuals participated in this experiment. Twenty young adults (age: 24.4 ± 1.1 years old, mean ± SD; 13 females, 7 males) and 20 older adult (age: 70.5 ± 5.6 years old; 16 females, 4 males) right-handed participants were tested using their right arm. All participants gave their informed consent to participate, as stipulated by the Ben-Gurion University Committee on Ethics.

The experimental procedure was identical to that described above.

The older group was invited back for a second meeting, during which their Mini-Mental test score was assessed. The second meeting occurred within 5–10 months of the first.

### Experiment 2 – Effects of Cognitive Load on Predictive Control

#### Participants

Twenty-three young adults (age: 23.4 ± 2.4 years old, mean ± SD; 13 females, 10 males; 21 right-handed, 2 left-handed) participants were tested using their dominant arm. All participants gave their informed consent to participate, as stipulated by the Ben-Gurion University Committee on Ethics.

##### Testing

The testing phase of Experiment 2 was identical to that of Experiment 1, except that participants were asked to count – either up or down – while performing the trials. In half of the trials they were counting up, in increments of one, from the number one. This was the No-Load (NL) condition. While cognitive abilities are needed in counting up in increments of one, it is assumed to be a minimally loading task, and is referred to as a NL condition for the purposes of the current experiment. In the other half of the trials, the participants were asked to count back, in increments of seven, from one of the following numbers: [100, 102, 104, 105, 106, and 109] [a serial subtraction task (Swanenburg et al., 2010; Muir-Hunter and Wittwer, 2016)]. This was the Cognitive-Load (CL) condition. In each CL trial they were asked to start counting back from a different number. The order of number presentation was counterbalanced. The CL and NL conditions alternated between trials (that is, each CL trial was followed by an NL trial and vice versa). The counting was monitored for accuracy, and every mistake was noted. No feedback on performance was given during the testing. Approximately half of the participants started with the CL condition, while the other participants started with the NL condition. Consequently, of the four INC trials, two were performed in the CL condition, and two in the NL condition, and the same was true for the four DEC trials.

### Data Analysis

**FIGURE 3:**
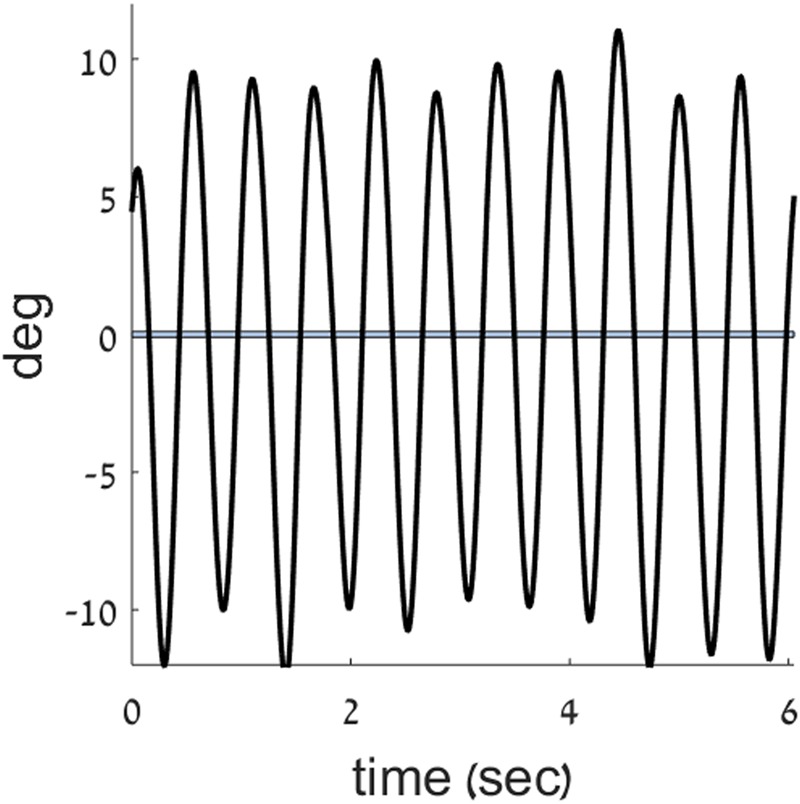
Zero crossings in the position trace. A position trace (black) from the current experiment is plotted, overlaid with a band around zero position, to mark the points of zero crossings in position.

The angular displacement of the forearm about the elbow joint, was filtered using a first order Butterworth filter with a cutoff frequency of 20 Hz. Trend was removed from the position data to reduce the effects of drift. This was achieved by removing the best straight-line fit from the angular position data. Data were analyzed with MATLAB^®^ software (Mathworks, Natick, MA, United States, v.9.1). To quantitatively determine the movement type performed by participants at each time point, we used the measure of harmonicity (Guiard, 1993, 1997; Buchanan et al., 2006; Hogan and Sternad, 2007; Levy-Tzedek et al., 2010, 2011b). The harmonicity index is a unitless number that is calculated for each movement half-cycle between two zero-crossings in the position record (see **Figure 3**). It is used in order to classify a movement as either highly rhythmic (when the harmonicity value is equal to 1, or close to it) or as containing discrete features. When a single peak in acceleration occurs in the half-cycle, the harmonicity value is set to one; when an inflection occurs in the half-cycle acceleration trace, the harmonicity is computed as the ratio of the minimum to the maximum absolute values of the acceleration within the given half-cycle; finally, if the acceleration trace within the half-cycle changes its sign, the harmonicity value is set to zero (Guiard, 1993, 1997). For instance, the harmonicity value calculated for the velocity trace shown in **Figure 1A** is 1, and the harmonicity value for the trace shown in **Figure 1B** is 0. The harmonicity index has previously been demonstrated to be a robust indicator of movement type (see, for example Levy-Tzedek et al., 2010).

Peaks in acceleration were found as follows: the jerk (the third derivative of the position) of the movement was calculated and was scanned for changes in sign. Each change in the sign of the jerk indicated a peak in acceleration occurred at that time point.

Harmonicity values were used to determine the frequency (**Fs**) at which a switch occurs between movement types (Levy-Tzedek et al., 2011b). In trials where the frequency of the movement continuously increased, the harmonicity values were scanned until the first instance of four consecutive half cycles had a harmonicity value above 0.5. That point was considered as the switch point between the two movement types. The cutoff value of harmonicity = 0.5 was chosen to comply with convention (Guiard, 1997; Buchanan et al., 2003, 2004, 2006; Levy-Tzedek et al., 2011b). Multiple movement cycles were examined to ensure that a switch point – rather than a momentary change in the nature of the movement – was indeed encountered.

If, in a given INC trial, there were no four consecutive half-cycles with a harmonicity value greater than 0.5, the trial was considered a no-switch (NS) trial.

The same method was applied for the trials in which the frequency was decreasing, but in this case checking for harmonicity *smaller* than 0.5. Similarly, if, in a given DEC trial, there were no four consecutive half-cycles with a harmonicity value smaller than 0.5, the trial was considered a NS trial.

This method for detecting the frequency at which the switch between movement types occurs has previously been validated by using an alternative method (a sigmoid function fit) for switch-point detection (Levy-Tzedek et al., 2011b).

#### Quantification of Predictive Control

We identified the frequencies at which participants switched between the discrete and rhythmic movement types described above (FsINC, the switch frequency on INC trials, in which the frequency increases), and between rhythmic and discrete movement types (FsDEC, the switch frequency on DEC trials, in which the frequency decreases). We tested whether they performed the switching in a predictive manner (that is, whether FsINC is lower than FsDEC), and compared the results of the two age groups, and the two conditions.

We use the term hysteresis (positive or reverse) to describe the pattern of switching between the two movement types – rhythmic and discrete. Hysteresis is the dependence of the state of a system on its history. In this case, the hysteresis stems from a switch that occurs at one frequency when the required movement frequency gradually increases, and at another frequency when the frequency gradually decreases. A positive hysteresis corresponds to FsINC that is higher than FsDEC, corresponding to reactive behavior, whereas a negative (or reverse) hysteresis is when FsINC is lower than FsDEC, corresponding to predictive behavior.

### Statistical Analysis

The Wilcoxon signed rank test for paired observations was used to compare the frequencies at which participants switched between the two movement types on INC vs. DEC trials. Data from participants who did not have a switch point at all in all four DEC trials or all four INC trials, or both, were not included in this analysis. Furthermore, a conservative approach was taken, and in cases where there was a single INC or a DEC trial with a switch point, out of the four trials, such that an average between two or more readings could not be taken, this participant’s data were not included in the statistical analysis, to avoid bias due to sampling error. A non-parametric test was chosen to eliminate the need for assumptions regarding population distributions required in parametric tests.

## Results

### Experiment 1 – Effects of Aging on Predictive Control

Both age groups performed on average about five practice trials during the training phase.

**FIGURE 4:**
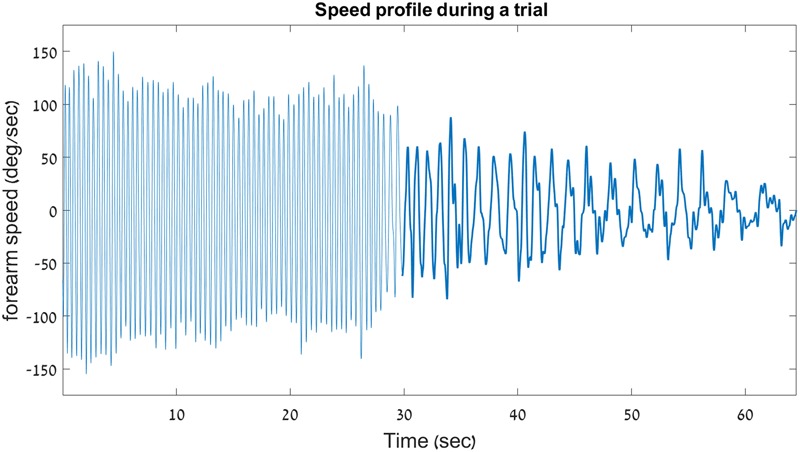
Sample velocity profile on a decreasing trial. Shown here is the velocity profile from a decreasing-frequency trial of a young participant in Experiment 1. For clarity, a thicker line is used to outline the discrete portion of the trial. This graph demonstrates the abrupt switching that occurs between the rhythmic and the discrete movement types during a single uninterrupted 66.5-s trial.

The velocity profile from two trials of a young participant – one INC trial and one DEC trial – are shown in **Figure 4**.

#### No-Switch (NS) Trials

Out of the 320 trials that were recorded and analyzed, 49 did not contain a switch point at all. That is, in those trials, participants maintained the same movement type (either rhythmic or discrete) for the entire duration of the trial, without switching to the other type, despite the requirement to switch in order to maintain the cursor within the enclosed area on the phase plane. There were 42 such trials in the older group (performed by 15 of the participants; out of these, 8 were INC trials, and 34 were DEC trials), and seven such trials in the younger age group (performed by five of the participants; out of these, four were INC trials, and three were DEC trials). These results suggest that for the older group switching from a rhythmic to a discrete movement type was more challenging than the reverse switch.

#### Switch Frequency (Fs)

**FIGURE 5:**
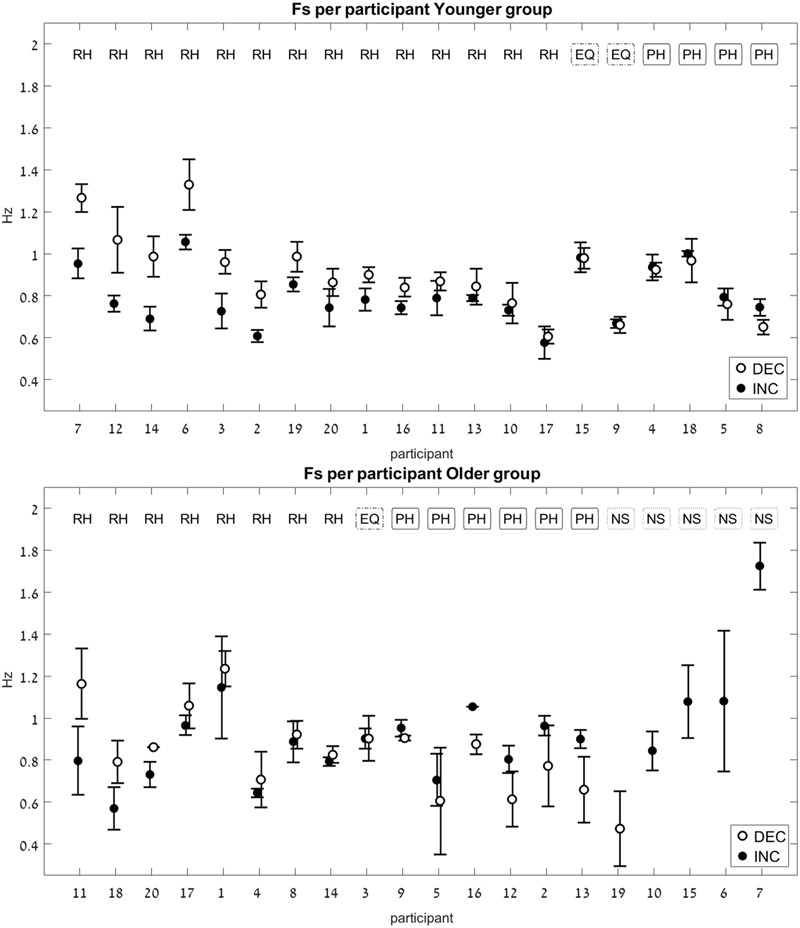
Switch-point frequency (Fs) in the two age groups (Experiment 1). Average values for Fs on the increasing-frequency trials (INC) are marked by a black circle, and those on the decreasing-frequency trials (DEC) are marked by a white circle. For each pair of INC-DEC results, two letters marked at the top indicate whether this pair shows a reverse-hysteresis pattern (RH), a positive hysteresis pattern (PH), the switch point frequency is equal at the INC and the DEC trials (EQ), or at least one of the INC/DEC blocks had no switch point at all (NS). For the sake of clarity, the results are grouped by pattern (RH/EQ/PH/NS), with the participant number on the *x*-axis corresponding to the chronological order in which participants were tested. **(Top)** Results for the young group; **(Bottom)** results for the older group. Error bars represent standard error.

For each participant, an average switch frequency was calculated for the INC and for the DEC trials. In cases where some of the INC or the DEC trials were NS (contained no switch), an average was obtained from the remaining trials, that did contain a switch. In the older group there were four participants for whom all four DEC trials had no switch point, and one participant for whom all four INC trials had no switch point (see **Figure 5**, bottom panel).

There was no evidence of a trend in the value of the switch point across trials for either age group (data not shown).

The pattern of the switch frequency for each participant was classified into one of three categories: reverse hysteresis (FsINC < FsDEC, noted as “RH”), positive hysteresis (FsINC > FsDEC, noted as “PH”), or no hysteresis (FsINC = FsDEC, noted as “equal,” or EQ). The values of the switch frequencies in the INC and the DEC trials were considered equal if the difference between them was 0.01 Hz or less in either direction.

#### Predictive Control

Only the data from participants who had switch points on both INC and DEC trials (even if not on all eight trials, but at least two repetitions in the INC trials and two in DEC trials) were included in the statistical analysis of the relative switch frequency between INC and DEC trials (20 young and 13 older adults). The paired analysis was performed separately for each age group.

The younger age group showed a significant difference between the FsINC and the FsDEC, with FsINC being lower than FsDEC on average (*p* = 0.004, see **Table 2**), as previously reported (Levy-Tzedek et al., 2011b). However, unlike the previously published results from young participants, where all participants exhibited a reverse-hysteresis switching pattern, in the current experiment there were individuals whose switching pattern deviated from the reverse-hysteresis pattern (see **Figure 5**, top panel). The current experiment tested a larger group of young participants (20 vs. 13), and the range of frequencies was shifted such that it contained more low frequencies and less high frequencies of movement, compared to the previous experiment (0.03–2.6 Hz vs. 0.042–3.9 Hz). This adjusted range was based on results from that previous experiment, showing that above 2.3 Hz there was no change in the harmonicity value. This change is not likely to account for the differences in the results between the two studies, since all switch-point frequencies in both studies fall within the two ranges that were chosen.

**Table 2:** Average switch frequencies (Fs) for the two experiments on the increasing-frequency trials (INC) and the decreasing-frequency trials (DEC).

	Experiment 1		Experiment 2	
	Young (*n* = 20)	Old (*n* = 20)	Young (*n* = 23)	
			With no cognitive load (NL)	With cognitive load (CL)
FsINC (Hz)	0.8 ± 0.1	0.9 ± 0.2	0.9 ± 0.2	0.8 ± 0.1
FsDEC (Hz)	0.9 ± 0.2	0.8 ± 0.2	1.0 ± 0.2	0.9 ± 0.2
95% confidence interval	[0.029, 0.18]	[-0.095, 0.093]	[0.038, 0.20]	[-0.049, 0.13]
*p*-value	**0.004**	0.9^∗^	**0.003**	0.4^∗∗^

*The *p*-values in bold denote that a significant difference was found between the FsINC and the FsDEC. ^∗^The statistical analysis was performed on the 13 old participants that had both an FsINC and an FsDEC, with at least two readings in each case. ^∗∗^The statistical analysis was performed on the 22 young participants that had both an FsINC and an FsDEC, with at least two readings in each case.*

There was no significant difference between the FsINC and the FsDEC in the older age group (*p* = 0.9).

The Mini-Mental test scores for the 20 older participants are shown in **Table 3**.

**Table 3:** Mini-Mental test scores.

Participant	Gender	Age^∗^	Years of education	Mini-Mental score	Switching pattern^∗∗^
1	F	70	16	30	RH
2	F	71	15	28	PH
3	M	65	24	29	EQ
4	F	75	14	28	RH
5	F	80	17	26	PH
6	M	78	21	27	NS
7	M	76	10	28	NS
8	F	69	15	29	RH
9	F	72	12	28	PH
10	F	64	10	30	NS
11	F	63	12	30	RH
12	F	68	15	28	PH
13	F	71	15	28	PH
14	F	69	10	22	RH
15	M	85	15	28	NS
16	F	64	15	28	PH
17	F	74	15	28	RH
18	F	69	9	27	RH
19	F	70	12	30	NS
20	F	71	10	29	RH

*^∗^The age at which the Mini-Mental test score was evaluated. ^∗∗^The letters here correspond to those in **Figure 5**. RH indicates a reverse hysteresis, PH indicates a positive hysteresis, EQ implies there was effectively no difference between the FsINC and FsDEC, and NS indicates there was no switch point in at least one of the sets (INC or DEC).*

Two of the older adults and three of the young adults reported at the end of the experiment that they felt there were two types of movement: a slow one and a fast one.

### Experiment 2 – Effects of Cognitive Load on Predictive Control

Participants performed on average about four practice trials during the training phase.

#### No-Switch Trials

**FIGURE 6:**
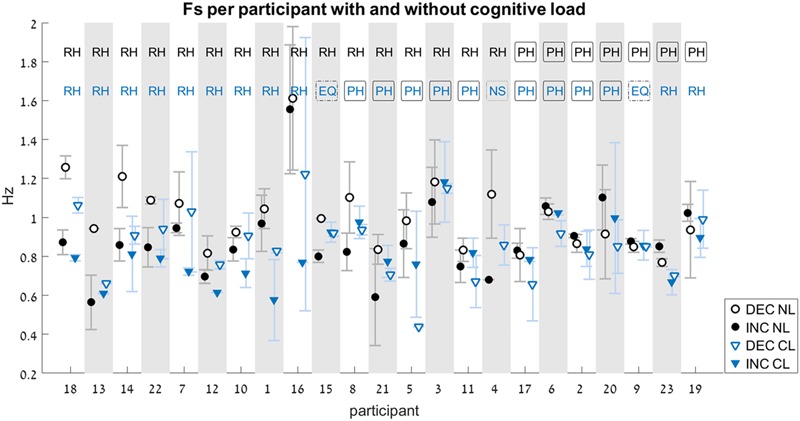
Switch-point frequency (Fs) with and without cognitive load (Experiment 2). Average values for Fs on the increasing-frequency trials (INC) are marked by filled shapes (circle or triangle), and those on the decreasing-frequency trials (DEC) are marked by an empty shapes. For each pair of INC-DEC results, two letters marked at the top indicate whether this pair shows a reverse-hysteresis pattern (**RH**), a positive hysteresis pattern (**PH**), the switch point frequency is equal at the INC and the DEC trials (**EQ**), or at least one of the INC/DEC blocks had no switch point at all (**NS**). For the sake of clarity, the results are grouped by pattern (RH/EQ/PH/NS), with the participant number on the x-axis corresponding to the chronological order in which participants were tested. Results from the No-Load condition (NL) are marked with black circles, while those from the Cognitive-Load condition (CL) are marked with blue triangles (similarly, the two-letter designation are black for the NL condition and blue for the CL condition). Error bars represent standard error.

Out of the 184 trials that were recorded and analyzed, eight did not contain a switch point at all. In those trials, participants maintained the same movement type (either rhythmic or discrete) for the entire duration of the trial, without switching to the other type. These were performed by six of the participants. Out of these, three were in the NL condition (one was an INC trial, and two were DEC trials), and five in the CL condition (four were INC trials, and one was a DEC trial). There was a single participant for whom there was no switch point in both INC trials under the CL condition (see **Figure 6**, participant # 4).

#### Predictive Control

Only the data from the 22 participants who had switch points on both INC and DEC trials were included in the statistical analysis of the relative switch frequency between INC and DEC trials.

In the NL condition, participants showed a significant difference between the FsINC and the FsDEC, with FsINC being lower than FsDEC on average (*p* = 0.003, see **Table 2**). However, the same participants did not show a significant difference between the FsINC and the FsDEC when exposed to a cognitive load (CL, *p* = 0.4). Sixteen participants showed a reverse-hysteresis switching pattern in the NL condition, as opposed to 11 participants in the CL condition (see **Figure 6**).

A total of 19 counting errors were made during the serial subtraction task, 10 of them during INC trials and nine during DEC trials.

Two of the young adults in Experiment 2 reported at the end of the experiment that they felt there were two types of movement, one that was slow and one that was fast.

## Discussion

In the current study, our goal was to study the effects of aging and of cognitive load on predictive control of movement. To that end, we employed a task that required an implicit (unaware) switch between two movement types. That switch can be made in a pro-active, anticipatory manner, or in a reactive manner. We found that both aging and cognitive loading impaired, on average, the ability to predictively control movement. In Experiment 1, which examined the effects of age on predictive control, less than half of the older participants were able to predictively control their movements [8 out of 20 (40%), compared with 14 out of 20 (70%) in the young group], as manifest in a reverse-hysteresis switching pattern. Four of the 20 older participants did not switch at all from high to low frequency movements, and one of the participants in this group did not switch at all from low to high frequency movements. This suggests that perhaps the difference we find between the age groups can be partly ascribed to a high cost of switching between motor plans for the older group (Hirsch et al., 2016). In Experiment 2, which examined the effects of cognitive load on predictive control, slightly less than half of the participants were able to predictively control their movements with a cognitively loading task [11 out of 23 (48%), compared with more than two-thirds (16 out of 23 (70%)] in the NL condition). One of the participants in this experiment did not switch at all from discrete to rhythmic movements when he was subject to a cognitive load (but not when there was no cognitive load).

### How Can an Implicit Task Be Affected by Cognitive Load or Cognitive Decline?

An implicit task is often defined as one where the ability to explicitly express “what” procedures are performed is diminished or non-existent (such as how to ride a bicycle) (Vidoni and Boyd, 2007), and thus a concurrent cognitive demand should not interfere with its performance. It may seem paradoxical to claim that a cognitive load, or cognitive decline, can affect the performance of an implicit task. However, a closer look at the literature reveals that many motor tasks contain both implicit and explicit components (several examples are reviewed in Reschechtko et al. (2016). Reschechtko et al. (2016) even showed that the same group of muscles, performing a single task, can have different output variabilities on instructed (explicit) vs. non-instructed (implicit) elements of the task. As a result, cognitive state can play a part in the outcome of motor tasks that contain both implicit and explicit components. Indeed, adaptive motor learning, which was once believed to depend solely on implicit memory, has been shown to decline with age as a function of explicit memory performance (Trewartha et al., 2014). Another example of this seemingly paradoxical combination is the effect of cognitive load on APAs. As discussed in the Introduction section, the studies on APAs showed that control of explicit elements (such as bending) is done separately from the control of implicit elements within the same movement (such as maintaining balance during the weight shift), supporting the dual-process hypothesis (Slijper et al., 2002). Indeed, studies on the effects of cognitive load on postural adjustments reveal degraded performance in both in young and in old participants (Shumway-Cook et al., 1997; Melzer et al., 2001). Thus, the current finding, that a cognitive load (and potentially a cognitive decline) affected performance on this task can be explained by recognizing that the task has both implicit and explicit components to it. These are, respectively: switching between motor tasks (rhythmic and discrete), and cyclically moving their forearm to control a cursor on the screen at changing speeds.

### Reverse Hysteresis as an Indication of Predictive Control of Movement

The hysteresis phenomenon is commonly found in nature, and can be described as a limited tendency to stay at a given state, despite a changing environment. It has the benefit of avoiding rapid oscillations between states around the switch point between these states, which may have an associated cost (Alexander, 1989). A cost of switching between tasks has been demonstrated in a wide variety of experiments involving both motor and cognitive tasks (e.g., Monsell, 2003; Hirsch et al., 2016).

Most examples found in nature are of classical hysteresis, while reverse hysteresis is less common. A plausible explanation for the reverse hysteresis pattern found in the current, as well as a previous study (Levy-Tzedek et al., 2011b), is that predictive control guides the early switching between movement types, in anticipation of the future. Predictive control is a form of control that incorporates a prediction of the future behavior of the system. The ability to identify a change in the environment and adapt to it in feed-forward manner, rather than rely on the inherently delayed feedback-based response is important. It can serve as a means to avoid injury, or even life-threatening situations (Porr and Worgotter, 2003; Gilbert and Wilson, 2007).

In both Experiments 1 and 2, we found that participants performed a mix of classical-hysteresis (positive hysteresis) and reverse-hysteresis switching patterns. This mix is found for young individuals when studying the walk-to-run (WR) and run-to-walk (RW) transitions in the lower limb. Experimental evidence showed either a classical hysteresis (e.g., Hreljac, 1995), revealed no difference between the WR and RW transitions speed (Prilutsky and Gregor, 2001), or showed reverse hysteresis (Getchell and Whitall, 2004). Diedrich and Warren (1995, 1998) reported individual behavior ranged from a significant classical hysteresis to a significant reverse hysteresis. Evidence for reverse hysteresis is found in Thorstensson and Roberthson (1987) as well, but significance values are not reported. Getchell and Whitall (2004) suggested that the reverse hysteresis phenomenon is the result of the effect of participants’ intention on the coordination dynamics, lending support to our predictive control hypothesis. Researchers have recently theorized that a classical hysteresis pattern emerges in gait patterns when participants are actively engaged in the task, and a reverse hysteresis pattern results when they are asked to evaluate how they would perform the task, without actually performing it (Abdolvahab and Carello, 2015). This theory is not supported by our upper-limb data. A study on arm movements, using an explicit timing cue, reported weak evidence of a classical hysteresis pattern in total movement time, manifest as a greater tendency to maintain a higher-frequency movement, even as the pace of the metronome slows down (Sternad et al., 2013). However, that study used different parameters for describing the arm movement at different frequencies, which preclude direct comparison with the current data.

Thus, data from both the upper and the lower limb are consistent with an interpretation that people often engage in predictive control of movement, and change their control strategy in preparation for a change in the environment, rather than perform this change only in feedback form, after the environmental change has taken place. This behavior can be advantageous, as it bypasses the inherent delays in feedback-based reactions to a changed environmental context.

Recent evidence from behavioral studies further supports this interpretation. Rosenbaum et al. (2014) have demonstrated in a series of experiments the propensity of participants to start a task early, even at the expense of extra physical effort. They termed this unexpected behavior “pre-crastination.” This can be envisaged as yet another form of “pre-acting.” That is, making a change to one’s motor plan in anticipation of future demands.

### Cognitive Load and Predictive Control

The results from Experiment 2 suggest that there is a cognitive component to the predictive control of movement. This result is supported by research showing that predictive control of saccadic eye movements is affected by a cognitive load (Lehtonen et al., 2012). The finding that both age and a cognitive load had a similar effect on the predictive control of movement might have suggested that potentially a decline in cognitive abilities underlies the decreased ability to predictively control movements with older age. While this is not reflected in the Mini-Mental scores of the participants in the current experiment, it would potentially be manifest in finer tests that examine executive control directly. The hypothesis that cognitive decline may impair predictive control should be examined in a separate study.

### Aging and Predictive Control

There are several changes that underlie aging, including changes in cognition and in motor-output control (Beauchet et al., 2007; Kilby et al., 2014; Sternäng et al., 2015) that affect activities of daily living, such as driving (Lodha et al., 2016). Can we identify the specific difficulties that lead to reduced performance? In one study, older adults have been found to not take advantage of color cues to predictively control their grip force when lifting an object (Cole and Rotella, 2002). Other work suggests that for older adults, the cost of switching between certain tasks is higher than for younger adults (Hirsch et al., 2016), which would suggest an inertial tendency (Rosenbaum et al., 2007) to maintain a motor plan without switching it, if possible. In other words, older adults appear to employ a more conservative switching strategy. This view is in line with other research suggesting that aging is accompanied by an increased reliance on slower feedback-based control of movement, as opposed to rapid feed-forward control (Bagesteiro and Sainburg, 2009).

Our findings further support these suggestions, in two ways: (1) more participants in the older group showed a positive-hysteresis pattern than in the younger group; and (2) some participants in the older group (and not in the young group, Experiment 1) failed to switch between movement types altogether, despite this being a requirement of the task. It suggests that, for the older adults, a high switching cost between motor plans (Monsell, 2003) leads to perseverance in performing the same type of movement (rhythmic or discrete) over longer extents of time.

And yet, we found that for 40% of the participants in the older age group were successful in predictively controlling their movements, and they displayed a reverse-hysteresis switching pattern. These results are in accordance with recently published data on APAs, showing that aging does not impair these preparatory adjustments (Plate et al., 2016), and with several studies on anticipatory control of grip force in older adults, showing relatively intact anticipatory control during simple grasp manipulations (for a review, see Diermayr et al., 2011). Together, these findings suggest that implicit predictive control of movement is not uniformly affected by age. Future studies should examine a potential correlation between cognitive function and the capacity for predictively controlling movement.

### Generalization of the Current Findings

As noted in the introduction section, we specifically chose a task that was previously unfamiliar to the participants, so that any training effects from past experience would not be a factor in task performance. Moreover, we employed a task that calls for an *implicit*, unaware switch between motor plans, to eliminate the process of a conscious decision making, and get at the basic pattern of predictively switching between motor plans. The task being different from standard activities of daily living, we can only speculate as to whether the results from the current study generalize to familiar, explicitly performed task switching in everyday life. The agreement between previous studies (e.g., Melzer et al., 2001; Diermayr et al., 2011; Trewartha et al., 2014; Hirsch et al., 2016; Plate et al., 2016) and the current results suggest that that would be the case.

## Author Contributions

SL-T designed and programmed the experiment, supervised the experimental procedure, analyzed the data and wrote the manuscript.

## Conflict of Interest Statement

The author declares that the research was conducted in the absence of any commercial or financial relationships that could be construed as a potential conflict of interest.
